# Puerarin inhibits hepatocellular carcinoma invasion and metastasis through miR-21-mediated PTEN/AKT signaling to suppress the epithelial-mesenchymal transition

**DOI:** 10.1590/1414-431X20198882

**Published:** 2020-03-31

**Authors:** Yuan Zhou, Ruifeng Xue, Jinglin Wang, Haozhen Ren

**Affiliations:** 1 Department of Hepatobiliary Surgery, Affiliated Drum Tower Hospital of Nanjing University Medical School, Nanjing, Jiangsu Province, China; 2 Department of Hepatobiliary Surgery, Nanjing Drum Tower Hospital Clinical College of Nanjing Medical University, Nanjing, Jiangsu Province, China

**Keywords:** Hepatocellular carcinoma, Metastasis, Epithelial-mesenchymal transition, PTEN, miR-21

## Abstract

Hepatocellular carcinoma (HCC) is one of the most common primary malignant tumors of the liver worldwide. Liver resection and transplantation are currently the only effective treatments; however, recurrence and metastasis rates are still high. Previous studies have shown that the epithelial-mesenchymal transition (EMT) is a key step in HCC invasion and metastasis. Inhibition of EMT has become a new therapeutic strategy for tumors. Recently, puerarin, a well-characterized component of traditional Chinese medicine, has been isolated from *Pueraria radix* and exerts positive effects on many diseases, particularly cancers. In this study, CCK-8, EdU immunofluorescence, colony formation, wound healing, and migration assays were used to detect the effects of puerarin on HCC cells. We further analyzed the relationship between puerarin and miR-21/PTEN/EMT markers in HCC cell lines. Our results showed that HCC cell proliferation, migration, invasion, tumor formation, and metastasis were reduced by puerarin *in vitro* and *in vivo*. Additionally, puerarin inhibited the EMT process of HCC by affecting the expression of Slug and Snail. Moreover, oncogenic miR-21 was inhibited by puerarin, coupled with an increase in the tumor suppressor gene PTEN. Increasing miR-21 expression or decreasing PTEN expression reversed the inhibition effects of puerarin in HCC. These data confirmed that puerarin affects HCC through the miR-21/PTEN/EMT regulatory axis. Overall, puerarin may represent a chemopreventive and/or chemotherapeutic agent for HCC treatment.

## Introduction

Across the globe, hepatocellular carcinoma (HCC) is still a major health problem, as it is the fifth most common tumor in men and the seventh in women (1). Even with the development of early diagnosis and treatments, the survival rate of HCC patients has not been notably improved in recent decades (2). Unrestricted cell proliferation and distal organ metastasis are the major obstacles to clinical cancer treatment (3,4). Therefore, new treatment strategies for HCC are urgently needed. Recently, increasing research on traditional Chinese medicine has shown good therapeutic effects against HCC (5).

Puerarin is a type of flavonoid extracted from the dried roots of *Pueraria lobata*. Puerarin may be an effective preventive compound against various nervous system diseases by regulating apoptosis and the inflammatory response (6). Puerarin inhibits the formation and activation of Nlrp3 inflammasome, which is induced by high glucose (7). In addition, puerarin exerts antioxidative effects and scavenges reactive oxygen species in cells. Previous experiments have shown that puerarin can also treat a variety of liver diseases, such as liver fibrosis and nonalcoholic fatty liver disease (8). The molecular mechanisms of the anti-tumor effect of puerarin have been determined in different types of cancers, such as colon cancer, breast cancer, and hepatocellular carcinoma (9,10). The results showed that high concentrations of puerarin can inhibit the growth of breast cancer, and induce apoptosis of HT-29 colon cancer cells. Although the anti-tumor effect of puerarin in HCC has been verified (11), the molecular mechanism of puerarin in HCC is still unclear (12).

Epithelial-mesenchymal transition (EMT) is an essential step during the progression of metastasis in the vast majority of cancers. In the EMT process, epithelial cells acquire a mesenchymal phenotype (13). During tumor invasion and metastasis, the abnormal activation and inactivation of numerous signaling pathways are observed and are responsible for regulating the EMT process (14). Previous studies have suggested that the activation of the AKT/GSK-3β signaling pathway promotes metastasis by regulating the EMT of cancer cells (15). Moreover, activated AKT can promote an increased expression of Snail through phosphorylating GSK-3β. As a transcriptional downregulator of E-cadherin, Snail further alleviates the EMT process in HCC (16). In addition to Snail, Slug is another significant regulatory protein of EMT. Zeb1 is a master regulator of EMT; its deregulation has been observed in multiple cancers, and it can be induced by Slug (17). Slug is also controlled by AKT/GSK-3β (16). Furthermore, PTEN (phosphate and tensin homolog), deleted on chromosome ten and a well-defined cancer suppressor, is controlled by miR-21 and sequentially inactivates AKT (18). miR-21 negatively correlates with prognosis in patients with HCC (19). A previous study showed that puerarin can block paraquat-induced oxidative stress by inhibiting miR-21 expression, leading to the attenuation of paraquat-induced lung fibrosis (20 [Bibr B21]). Based on previous research, we hypothesized that puerarin may interfere with HCC progression by inhibiting miR-21.

In this study, we aimed to investigate the anti-tumor function of puerarin in HCC treatment and reveal its specific mechanism.

## Material and Methods

### Animals

Male BALB/c nu/nu mice (4–5 weeks old) were purchased from Shanghai Institute of Material Medicine, Chinese Academy of Science and housed under specific pathogen-free conditions. All animals received humane care according to the criteria outlined in the “Guide for the Care and Use of Laboratory Animals” prepared by the National Academy of Sciences and published by the National Institutes of Health (NIH publication 86–23, revised 1985). All animal procedures were approved by the Animal Research Ethics Committee of Nanjing Medical University.

### Animal studies

For examining the roles of puerarin in HCC treatment, 1×10^7^ Bel-7402 cells or Huh7 cells in 0.2 mL of sterile PBS were injected subcutaneously into the right side of each male nude mouse. After 45 days, the mice were sacrificed and the tumors were excised and weighed. The tumor nodules formed on the liver surfaces were counted.

### HCC cell lines

All cell lines (Bel-7402, Huh7, and L02) were purchased from the Cell Bank of Type Culture Collection of the Chinese Academy of Sciences (Shanghai Institute of Cell Biology). Bel-7402 and L02 cells were cultured in 1640 medium (Gibco, Australia), and Huh7 cells were cultured in DMEM medium (Gibco). All cells were cultured with 10% fetal calf serum (Gibco), and 1% penicillin/streptomycin (Gibco). All cells were maintained in a humidified atmosphere containing 5% CO_2_ at 37°C.

### Reagents and antibodies

Puerarin (HY-N0145) and PTEN inhibitor (VO-Ohpic, HY-13074) were purchased from MEC (USA); miR-21 mimic was purchased from RIBOBIO (miR10000530-1-5, China). The following antibodies were purchased from Cell Signaling Technology (USA): E-cadherin (3195, 1:1,000), N-cadherin (13116, 1:1,000), Vimentin (5741, 1:1,000), Snail (3879, 1:1,000), Slug (9585, 2:1,000), PTEN (7960, 1:1,000), PDK1 (3062, 1:1,000), phospho-PDK1 (Ser241) (3438, 1:1,000), phospho-Akt (Ser473) (4060, 1:1,000), AKT (4691, 1:1,000), GSK-3β (12456, 1:1,000), phospho-GSK-3β (Ser9)(5558, 1:1,000), Ki67 (9027, 1:400), and GAPDH (5174, 1:1,000). All reagents and antibodies were used according to the manufacturer's instructions.

### Cell viability assay (CCK-8)

Cell proliferation was assessed by CCK-8 according to manufacturer's instructions. Briefly, cells were seeded into 96-well plates at a density of 5×10^3^/well. The cells were cultured with different treatments for 6, 12, 24, and 48 h. Subsequently, a diluted CCK-8 reaction solution was added into each well, followed by incubation at 37°C for 2–4 h. Then, the absorbance at a wavelength of 460 nm was determined to estimate cell viability.

### Invasion assays

The transwell cell invasion assays were performed in 8-μm porosity BD BioCoat Matrigel Invasion Chambers (BD Biosciences, USA), as described previously (21). HCC cells (2×10^4^) in serum-free medium were seeded onto Matrigel-coated filters in the upper chambers. Medium containing 15% FBS was added to the lower chambers. After 48 h of incubation, the cells on the upper surface of the filters were removed with a cotton swab, and the filters were fixed with 100% methanol and stained with Giemsa. The invasive ability of HCC cells was calculated as the mean number of cells in all fields and expressed as the relative ratio compared with control cells.

### Wound-healing assay

Cells were seeded into six-well plates and cultured at 37°C with 5% CO_2_ for 24 h. A wound was created every 1 cm in each well by scratching with a 200-μL pipette tip. The cells were washed twice with phosphate-buffered saline (PBS), and then 2 mL of conditioned culture medium with different drugs was added to each well. Wound closure was monitored at 0, 24, and 48 h, and representative scratch lines were imaged using an inverted microscope equipped with a Nikon (Japan) camera.

### Western blotting

Whole-cell protein extracts were homogenized in lysis buffer and centrifuged at 140,000 *g* for 15 min at 4^o^C. Bicinchoninic acid (BCA) assay was performed to measure the protein concentrations. After immunoblotting, the proteins were transferred to nitrocellulose filters that were subsequently incubated with specific antibodies. The immunocomplexes were incubated with a fluorescein-conjugated secondary antibody, and then detected using an Odyssey fluorescence scanner (Li-Cor, USA). The greyscale value was measured using Image J (NIH, USA).

### 5-Ethynyl-20-deoxyuridine (EdU) assay

The EdU assay kit (Keygene, China) was used to evaluate the proliferative activity of cells. HCC cells were cultured in 24-well plates with puerarin or control medium for 48 h. The cells were incubated with 200 µL of EdU (50 mM) at 37°C for 2 h and then fixed in 4% paraformaldehyde for 30 min. The cells were permeabilized with 0.5% Triton X-100 for 10 min, followed by incubation with 200 µL of Apollo reaction liquid for 30 min at room temperature. Subsequently, 200 µL of Hoechst 33342 was added to each well, followed by incubation for 30 min. The images were then acquired under a fluorescence microscope (Leica, Germany) at 200× magnification, and the number of proliferative cells in five randomly selected fields was calculated for each sample.

### Immunohistochemistry

Immunohistochemistry (IHC) of HCC samples was performed as previously described (22) with incubation with antibodies against Ki67, N-cadherin, Snail, and Slug, all from Cell Signaling Technology (USA). IHC results were scored according to Table 1.

**Table 1:** The immunohistochemical (IHC) scoring criterion is shown below.

Score	Staining intensity	Proportion of positive cells
0	Negative	<5%
1	Weak	5−25%
2	Moderate	26−50%
3	Strong	51−75%
4	NA	>75%

Staining score = staining intensity × proportion of positive cells (0−12). IHC results of miRNA-21 were scored as follows: 0, staining value 0−3; 1, staining value 4−6; 2, staining value 7−9; 3, staining value 10−12. NA: not applied. IHC results were scored by two experienced pathologists.

### Immunofluorescence (IF) assay

The IF assay was performed according to previously established protocols (23). Cells were seeded into 24-well plates and fixed with 4% paraformaldehyde after 48-h treatment of puerarin or control. Fixed cells were stained with vimentin, Snail, and Slug, followed by incubation with FITC-conjugated anti-mouse IgG and FITC-conjugated anti-rabbit IgG (Cell Signaling Technology). Representative images were acquired by fluorescence microscopy (Leica), and the data were processed via ImagePro Plus (USA).

### RNA extraction and quantitative real-time PCR

RNA was extracted from cells with TRIzol^TM^ reagent (Life Technologies, USA) according to the manufacturer's instructions. Briefly, cells in six-well plate were homogenized in 1 mL TRIzol reagent at room temperature. Then, 200 μL of trichloromethane was added and mixed thoroughly, followed by centrifugation at 12,000 *g* for 15 min at 4°C. The upper clear phase was collected and 500 μL of isopropanol was added, followed by centrifugation at 12,000 *g* for 10 min at 4°C. The pellet was washed with 75% ethanol. The RNA was re-suspended in 30 μL of RNase-free DEPC-water and stored at −80°C. Reverse transcription was performed with PrimeScript^TM^ RT Master Mix (Takara, Japan) according to the manufacturer's instructions. qRT-PCR was performed using TB GreenTM Premix Ex TaqTM (Takara) and ABI PRISM 7500 real-time PCR System (Applied Biosystems, USA). Primers used for qPCR are shown in Table 2. The relative expression levels of mRNA were calculated with the 2^-ΔΔCt^ method. The housekeeping gene GAPDH was used as the internal normalization control for the mRNA.

**Table 2:** Primers for mRNA gene and miR-21 mimic sequence.

Gene	Forward primer	Reverse primer
E-cadherin	AACGAGGGCATTCTGAAAACA	CACTGTCACGTGCAGAATGTACTG
N-cadherin	ACCTGAACGACTGGGGGCCA	TGCCAAAGCCTCCAGCAAGCA
Vimentin	CCATCAACACCGAGTTCAAGAA	GGCGAAGCGGTCATTCAG
Slug	ACGCCCAGCTACCCAATG	CGCCCCAAAGATGAGGAGTA
Snail	CACTATGCCGCGCTCTTTC	GGTCGTAGGGCTGCTGGAA
GAPDH	GGAGCGAGATCCCTCCAAAAT	GGCTGTTGTCATACTTCTCATGG
miR-21	TTTTGTTTTTGCTGGTCTTAG	AGCAGACAGTCAGGCAGGAT
U6	GCTTCGGCAGCACATATACTAAAAT	CGCTTCACGAATTTGCGTGTCAT
miR-21 mimic	5′-AUCGAAUAGUCUGACUACAACU-3′	

Mimic is a single chain sequence.

The miRNeasy Mini Kit (Qiagen, Germany) was used to extract miRNA from cells according to the product protocol. Total RNA was stored at −80°C for subsequent experiments. Reverse transcription and qRT-PCR for exosomal miRNA and internal reference U6 were performed using miRNA RT-PCR Quantitation Kit (Qiagen) according to the manufacturer's instructions. The reactions were initiated with denaturation at 95°C for 3 min, followed by 40 cycles of 95°C for 15 s and 62°C for 34 s. The relative expression levels of miRNA were calculated with 2^-ΔΔCt^.

### Statistical analysis

Data analysis was performed using SPSS software version 16 (USA). Each experiment was performed as least in triplicate, and all results are reported as means±SD. The chi-squared test and Student's *t*-test were used to assess statistical significance. Kaplan-Meier analysis and log-rank tests were applied for survival analysis. A value of P<0.05 was considered significant.

## Results

### Puerarin inhibited HCC cell proliferation and tumor formation

**Figure 1:**
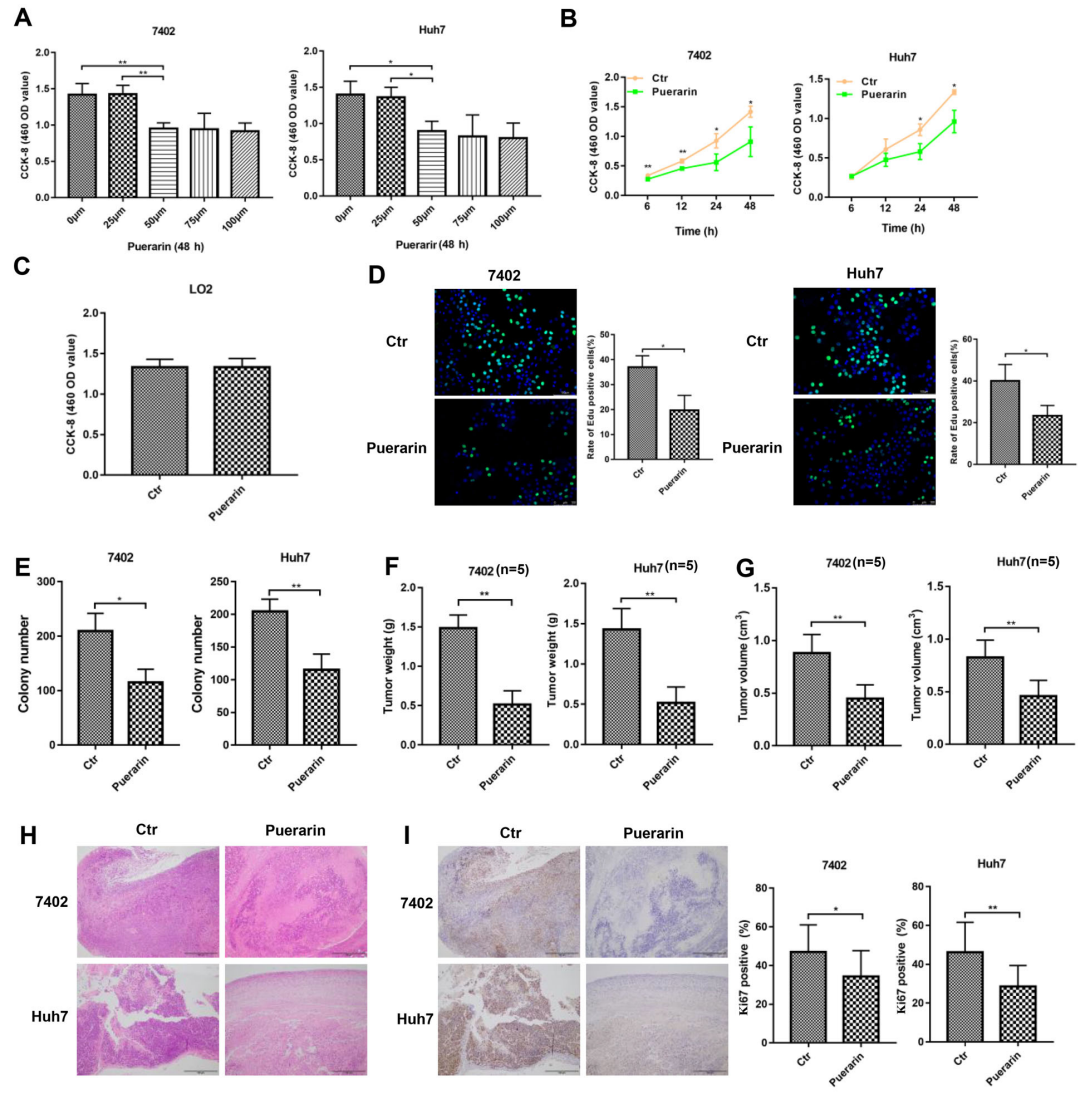
Effects of puerarin on hepatocellular carcinoma (HCC) proliferation and growth. **A** and **B**, CCK-8 assays of HCC cells treated by puerarin at different concentrations and time points. **C**, CCK-8 assays of L02 cells treated with puerarin at the concentration of 50 nM. **D** and **E**, Edu assay and liquid colony formation analysis of HCC cells were conducted to detect the anti-tumor effects of puerarin (magnification bar: 100 μm). **F** and **G**, Colony number and tumor weight of CCE cells treated with puerarin or control. **H**, H&E staining was performed on serial sections of mouse tumors induced from HCC cells (magnification bar: 100 μm). **I** Immunohistochemistry analysis of Ki67 expression and qualification of Ki67-positive cells in mouse tumors (magnification bar: 100 μm). At least three independent experiments with similar results were done. Data are reported as means±SD. *P<0.05, **P<0.01 (*t*-test). Ctr: control.

**Figure 2:**
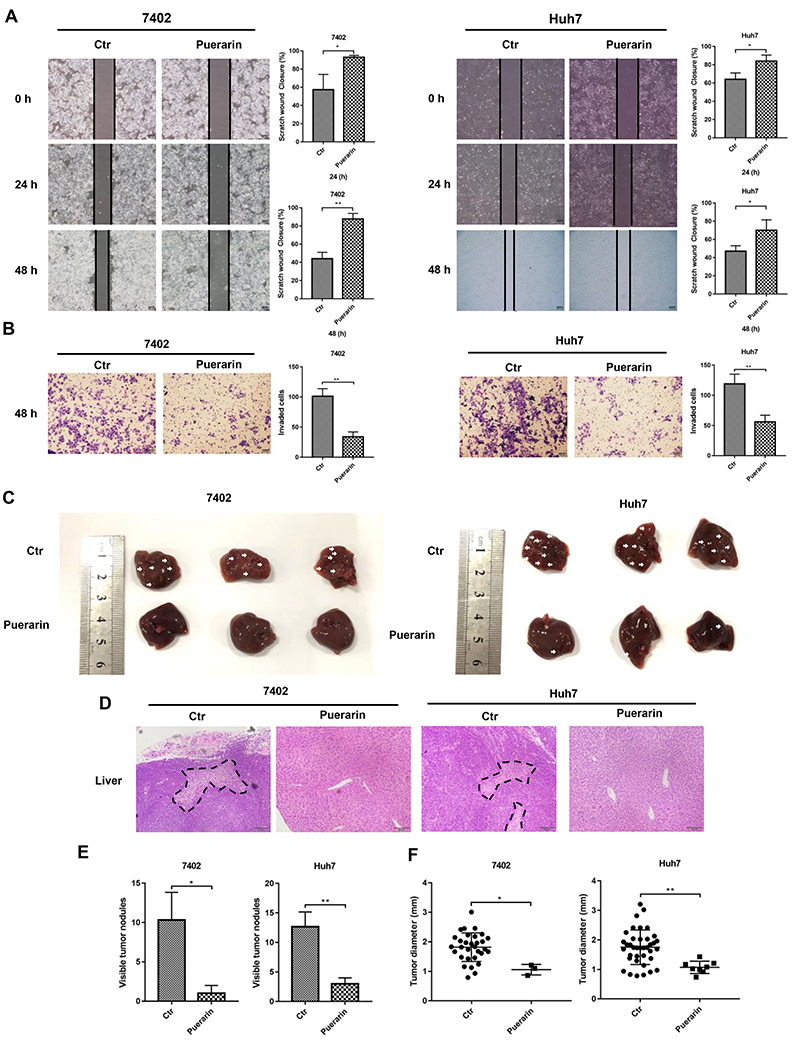
Puerarin inhibited hepatocellular carcinoma (HCC) cell migration and invasion. **A**, Wound-healing assays were performed at indicated times following initiation of the scratch in puerarin-treated HCC cells. **B**, Effect of puerarin on cell invasion through Matrigel assay (magnification bar: 100 μm). **C**, Images of metastatic liver nodules in nude mice. The arrows indicate the metastatic tumor on the surface of the liver. **D**, H&E staining was performed on serial sections of the liver tissue (magnification bar: 100 μm) Dotted lines indicate tumor extent. The number (**E**) and the diameter (**F**) of nodules were quantified in liver of nude mice. Data are reported as means±SD. *P<0.05, **P<0.01, Student’s *t*-test. Ctr: control.

To detect the inhibition effect of puerarin in HCCs, different concentrations of puerarin were added to the HCC cell (7402 and Huh7) culture medium according to previous studies (24). The results showed that HCC cell growth was inhibited by puerarin at different concentrations (from 25 to 100 mM), and puerarin showed effective inhibition at the concentration of 50 mM for 12, 24, and 48 h (Figure 1A and B). According to these results, we chose 50 mM as the effective concentration for HCC treatment. As expected, this concentration of puerarin had no effect on L02 cells, a normal liver cell line (Figure 2C). To further verify the anti-effect of puerarin in HCC, EdU and clone formation assays were conducted. Consistent with previous results, puerarin decreased DNA replication and clonal formation in HCC cells (Figure 1D and E). To further examine the anti-tumor capability of puerarin *in vivo*, puerarin was administered to mice with HCC subcutaneous tumor. All experimental animals were randomly divided into two groups: control group and puerarin group. The mice in the puerarin group (n=6) were injected with puerarin through the tail vein (40 mg/kg) every other day, while the mice (n=6) in the control group received the same volume injection of phosphate-buffered saline (PBS) (25). Within 45 days, there was no significant difference in the weight of the mice. However, the puerarin group showed reduced tumor weight and smaller tumor compared with those of the control group (Figure 1F and G). Less disordered cell arrays were observed in the puerarin group by H&E staining (Figure 1H). HCC cell proliferation was inhibited by puerarin as well, as determined through Ki67 staining (Figure 1I). The above results demonstrated that puerarin can inhibit proliferation *in vitro* and tumor formation *in vivo*.

### HCC cell metastasis was suppressed by puerarin

In Figure 2A, the migration of cells on the edge of the scratched area was inhibited by puerarin. Similarly, transwell assays revealed that puerarin reduced HCC cell invasion (Figure 2B). Thus, puerarin possesses the ability to reduce cell migration and invasion. We further verified the above phenomena through *in vivo* experiments. Six weeks after puerarin treatment, we calculated the number of metastatic nodules on the liver surface (Figure 2C). H&E staining was used to demonstrate that these nodules were metastatic HCC (Figure 2D). Increased numbers and larger diameter metastatic nodules were observed in the control group. However, the mice in the puerarin group had fewer liver metastatic nodules and the nodules had a smaller diameter (Figure 2D and E). Furthermore, puerarin did not damage the normal liver, with no obvious morphological changes in normal liver tissues (Figure 2D). To summarize the above results, we found that HCC cell invasion and metastasis were inhibited by puerarin.

### Puerarin inhibited HCC epithelial-mesenchymal transition

**Figure 3:**
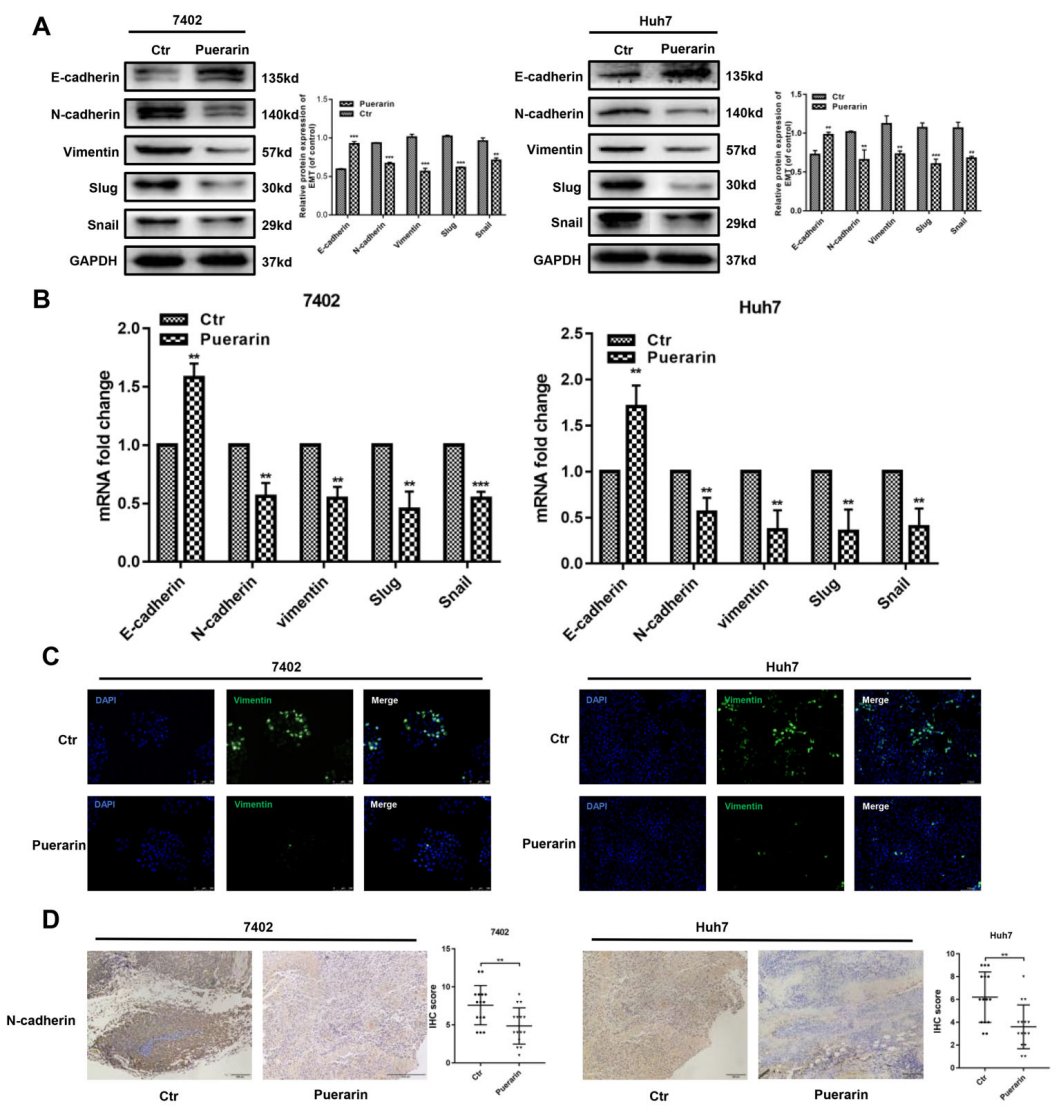
Effect of puerarin on epithelial-mesenchymal transition (EMT) genes. **A**, Western blotting analysis and **B**, qRT-PCR analysis was used to detect the expression of EMT markers in puerarin-treated hepatocellular carcinoma (HCC) cells. **C**, Immunofluorescence analysis of vimentin expression (magnification bars: 100 μm). **D**, Immunohistochemistry analysis (IHC) of N-cadherin expression was performed on hepatocellular tumors (magnification bars: 100 μm). Statistical analyses of the staining intensity of N-cadherin between different groups are shown. Data are reported as means±SD. **P<0.01, ***P<0.001 (*t*-test). Ctr: control.

Western blotting and qRT-PCR assays were used to detect the expression levels of EMT markers. Results showed that puerarin induced the expression of E-cadherin (an epithelial marker), coupled with low expression of vimentin and N-cadherin (two mesenchymal markers) in HCC cells (Figure 3A). qRT-PCR further indicated that puerarin inhibited EMT in HCC cell lines (Figure 3B). Immunofluorescence staining confirmed the inhibition of vimentin expression by puerarin (Figure 3C). We next detected the expression of N-cadherin in subcutaneous xenograft mouse models (26). Compared with the control group, the IHC staining of N-cadherin was significantly lighter in the puerarin group (Figure 3C).

**Figure 4:**
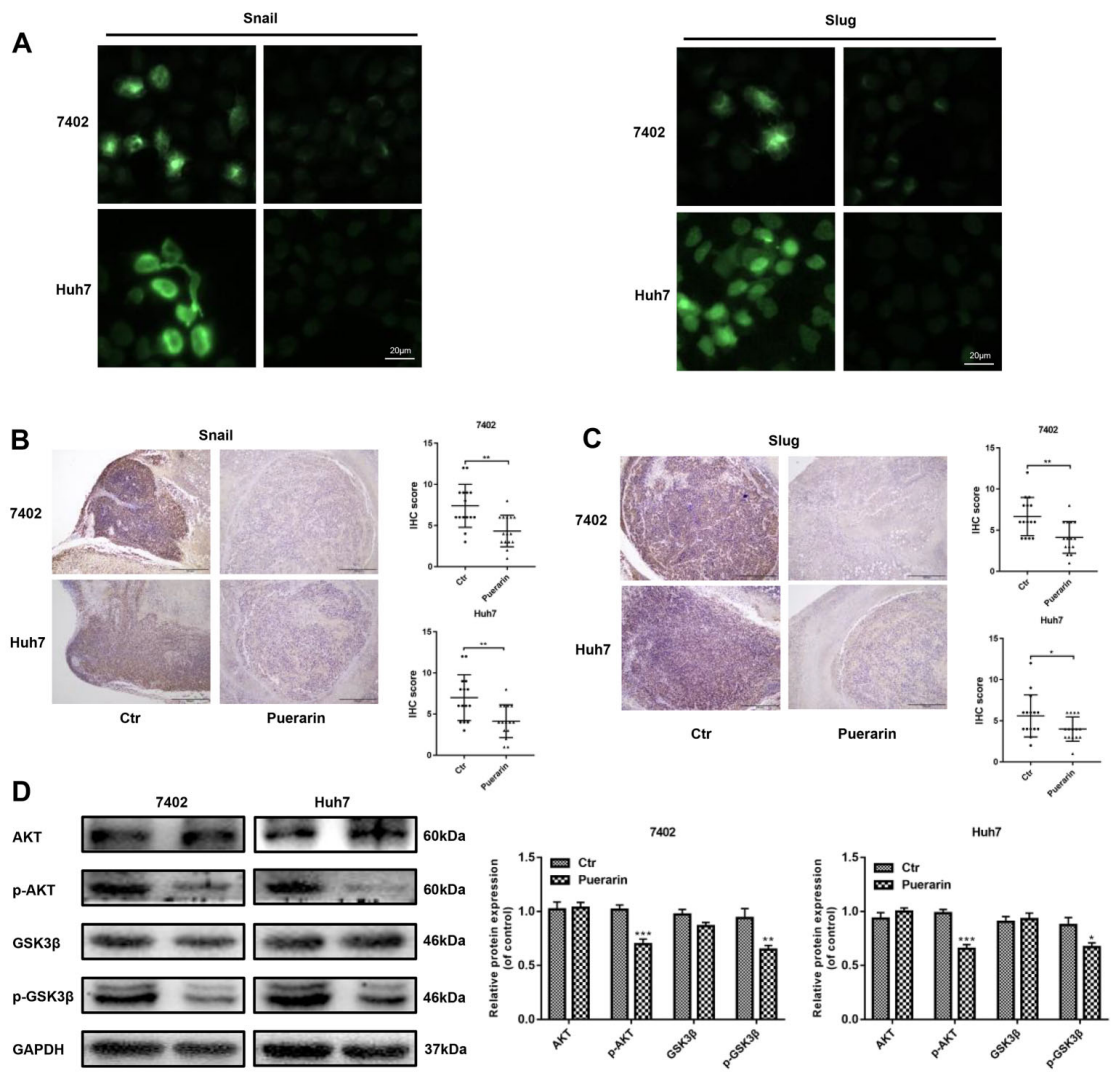
Effect of puerarin on epithelial-mesenchymal transition (EMT) regulators. **A**, Immunofluorescent staining was used to detect EMT regulator (Snail and Slug) expression in hepatocellular carcinoma (HCC) cells (magnification bars: 20 μm). **B** and **C**, Immunohistochemistry analysis (IHC) of Snail and Slug expression was performed on the same hepatocellular subcutaneous xenograft (magnification bars: 100 μm). **D**, Western blotting confirmed the expression change of EMT regulators in HCC cells. Data are reported as means±SD.*P<0.05, **P<0.01, ***P<0.001 compared to control (*t*-test). Ctr: control.

We further detected the underlying mechanism of the puerarin effect on EMT genes. Western blotting and qRT-PCR assays were used to explore the expression of Snail and Slug, two EMT-related transcription factors. The results showed that the protein expression and mRNA expression of Snail and Slug were decreased by puerarin (Figure 3A and B). Immunofluorescence of Snail and Slug further verified the above results (Figure 4A). Consistent with these findings, Snail and Slug expressions were analyzed in subcutaneous xenograft mouse HCC models by IHC staining. Low levels of Snail and Slug were primarily observed in the puerarin group (Figure 4B and C). Intriguingly, upstream phosphorylated AKT and GSK-3β were downregulated in puerarin-treated HCC cells (Figure 4D). Therefore, puerarin efficiently inhibited Slug and Snail expression.

### Puerarin regulated miR-21, enhanced PTEN expression, and subsequently decreased HCC progression

**Figure 5:**
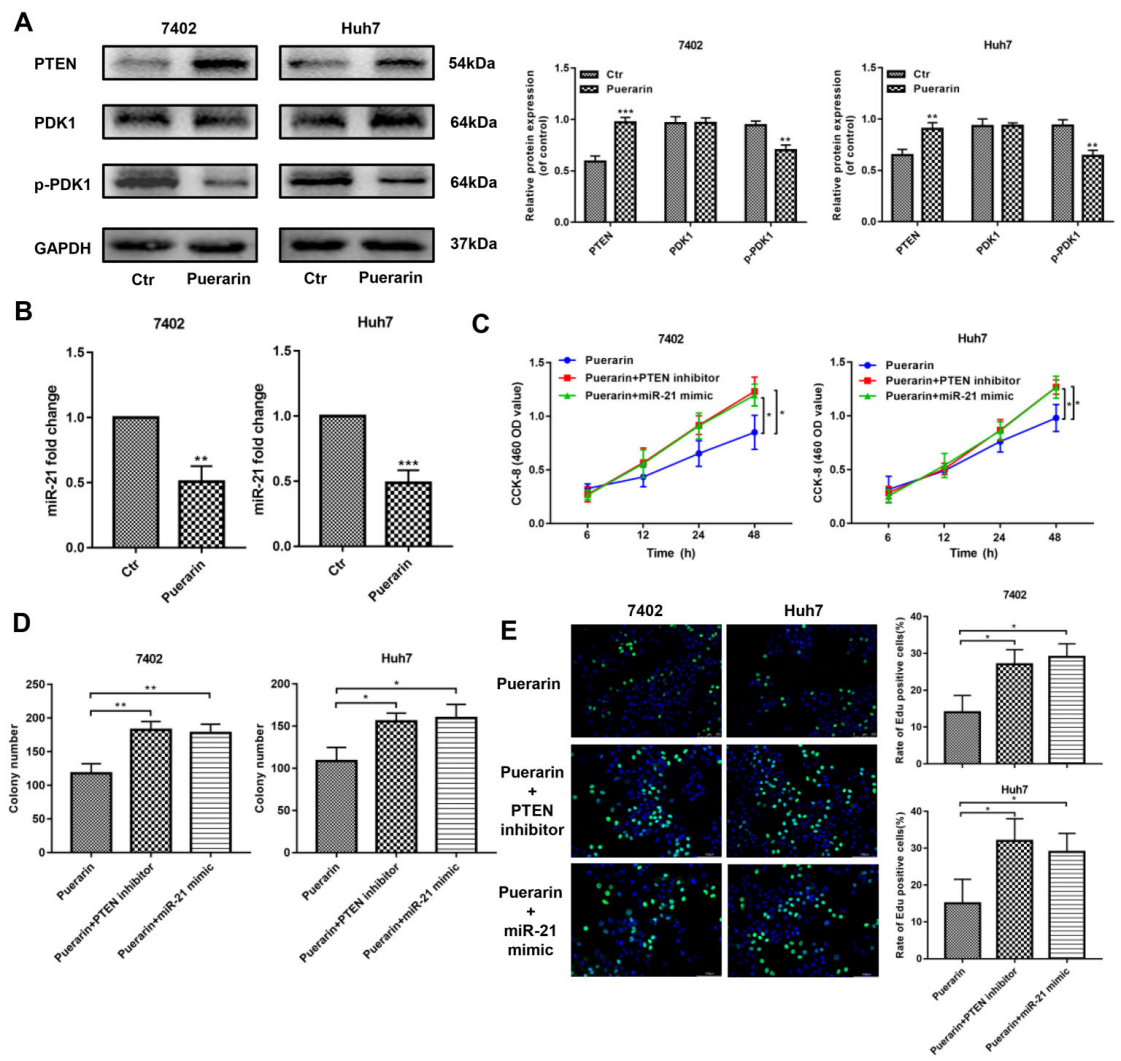
miR-21 mimic and phosphate and tension homolog (PTEN) inhibitor reversed the anti-tumor effects of puerarin in hepatocellular carcinoma (HCC) cells. **A**, Western blotting analysis was used to detect the PTEN expression and its downstream protein expression. **B**, qRT-PCR analysis confirmed the expression change of miR-21 in puerarin treated HCC cells. **C**, CCK-8 assays of treated HCC cells with miR-21 mimic and PTEN inhibitor at different time points. **D** and **E**, Liquid colony formation analysis and EDU assay of miR-21-infected and PTEN-inhibited HCC cells under the treatment of puerarin (magnification bars: 100 μm). Data are reported as means±SD. *P<0.05; **P<0.01. ***P<0.001 (*t*-test). Ctr: control.

As PTEN is a targeted regulatory protein upstream of AKT, we then detected PTEN expression in puerarin-treated HCC cells. As shown in Figure 5A, puerarin increased the protein level of PTEN, coupled with the low phosphorylation of PDK1 (3-phosphoinositide-dependent kinase 1), the downstream substrate of PTEN (27). At the same time, we observed that the expression level of PTEN was significantly inhibited by mi-21 mimic intervention, which also indicated that mi-21 regulated PTEN. Furthermore, miR-21, a well-known regulator of PTEN (28), was inhibited in puerarin-treated HCC cells (Figure 5B). These data indicated that puerarin may negatively regulate HCC through the miR-21/PTEN pathway.

**Figure 6:**
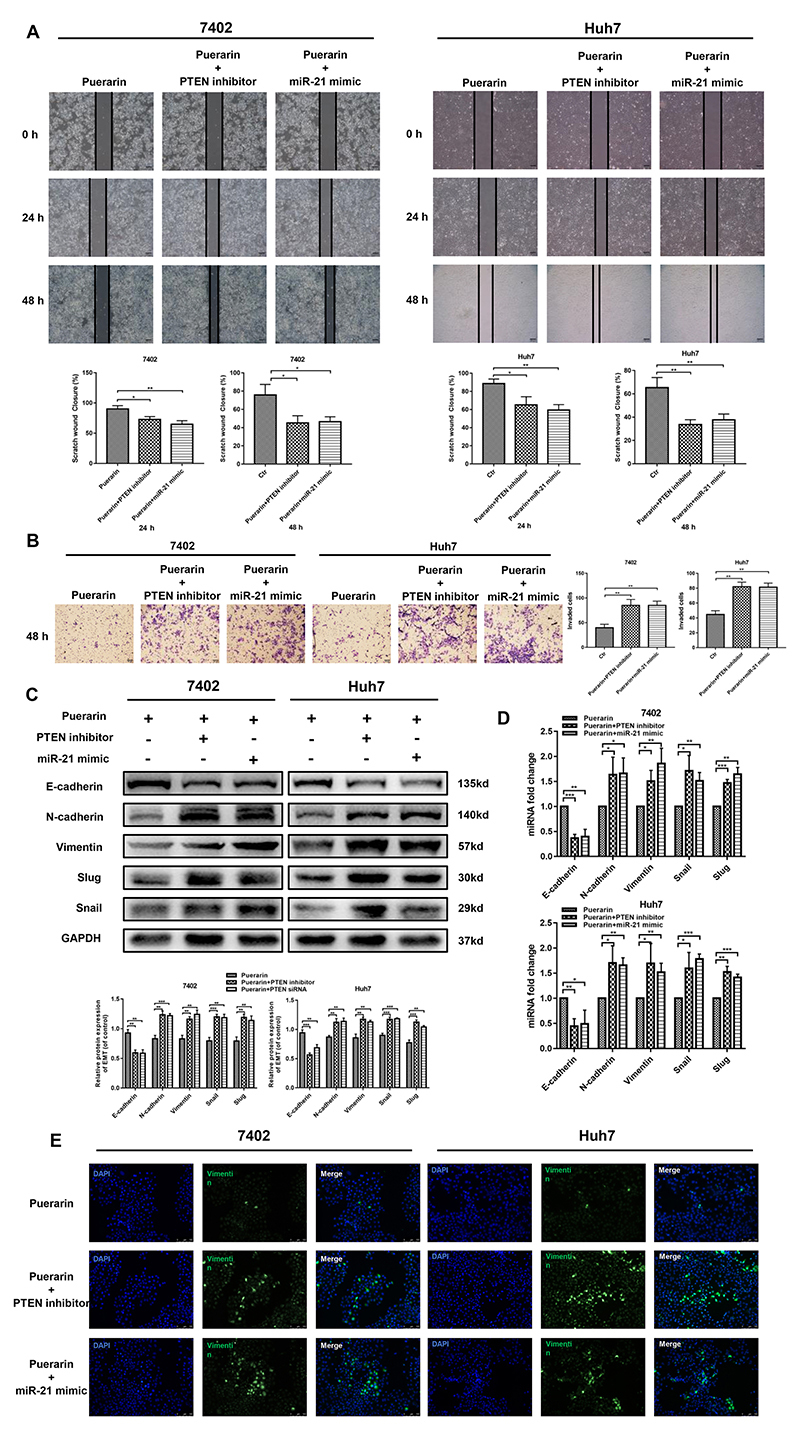
miR-21 mimic and PTEN inhibitor reversed the puerarin-inhibited invasion and metastasis. **A** and **B**, Representative images of wound-healing assay and corresponding statistical analysis (magnification bars: 100 μm). Overexpression of miR-21 and PTEN inhibitor markedly reversed cell motility, which was inhibited by puerarin. **C** and **D**, Expressions of E-cadherin, N-cadherin, vimentin, snail, and slug were assessed by western blotting and qRT-PCR after overexpressing of miR-21 and inhibition of PTEN. **E**, Immunofluorescence analysis of vimentin expression (magnification bars: 100 μm). Data are reported as means±SD from three independent experiments. *P<0.05; **P<0.01, ***P<0.001 (*t*-test). Ctr: control.

Next, we used a miR-21 mimic and a PTEN inhibitor (VO-Ohpic, 50 nM, according to the manufacturer's instructions) to verify changes in miR-21 and PTEN in puerarin-inhibited HCC development (29). The results showed that miR-21 mimic and PTEN inhibitor effectively rescued the inhibited cell proliferation (Figure 5C to E), migration, and invasion (Figure 6A and B) of HCC cells. Additionally, the results of western blotting and qRT-PCR suggested that the miR-21 mimic and PTEN inhibitor could restore the EMT progression of HCC cells that was inhibited by puerarin; the miR-21 mimic and PTEN inhibitor restored the downregulation of E-cadherin and upregulation of N-cadherin and vimentin (Figure 6C and D). These findings were supported by the results of vimentin immunofluorescence assays (Figure 6E). Collectively, the inhibition effects of puerarin depended on the regulation of the miR-21/PTEN axis.

## Discussion

Hepatocarcinogenesis is a multifactorial process that is associated with multiple genetic alterations involving the overexpression of oncogenes and the abnormal silencing of tumor suppressor genes (30). At present, despite continuous improvements in treatment methods, the high recurrence rates and development of metastasis still hamper the outcome of HCC patients (31). Recent studies have shown that EMT is a significant initiation step for cancer invasion (32). When EMT occurs in tumor cells, obvious changes in the expression level of EMT markers can be detected: E-cadherin is downregulated, while vimentin and N-cadherin are upregulated (33). Among the three markers, the reduced expression of E-cadherin is responsible for the EMT process in cancers (34). Several proteins have been identified as downregulators of E-cadherin expression, including Snail and Slug (34). In addition, activation of the AKT/GSK-3β pathway leads to EMT in multiple cancers and is responsible for the upregulation of Snail and Slug (35). Therefore, the molecular targets in the regulatory pathway of EMT have received great attention. Interventions based on regulating EMT associated biological factors may be an effective treatment strategy for HCC treatment.

*Pueraria radix* contains fairly high amounts of flavonoids, and puerarin is the most abundant one. Previous studies have verified its beneficial therapeutic effects on cardiovascular diseases and neurological diseases (36,37). Recently, research has suggested that high concentrations of puerarin can inhibit human breast cancer cell growth (38). Moreover, recent experiments have shown that puerarin has an inhibitory effect on many tumors, including HCC (39,40). Although previous studies have focused on cell proliferation and apoptosis, there has been no in-depth study on tumor invasion and metastasis. In our experiments, we observed that puerarin inhibited HCC cell proliferation at an effective concentration of 50 nM. Considering that invasion is the main cause of cancer metastasis, wound healing and transwell assays were used to determine that puerarin suppressed HCC cell migration and invasion *in vitro*. We further detected the puerarin inhibition of liver metastasis in an HCC xenograft model. Based on the anti-tumor function of puerarin, we proposed that puerarin inhibits HCC metastasis by regulating the EMT process and affecting the expression of EMT markers. We found that puerarin increased the expression of epithelial markers at the transcriptional and protein levels in HCC cells, coupled with decreasing the expression of mesenchymal markers. Immunofluorescence analysis of vimentin in puerarin-treated HCC cells and IHC staining of N-cadherin in mice HCC subcutaneous xenograft tumors confirmed the inhibition effect of puerarin. Under puerarin treatment, the metastatic ability of HCC cells was reduced. The tumor sizes were smaller and there were fewer nodules in the livers of nude mice in the puerarin group. Moreover, AKT phosphorylation was inhibited by puerarin, resulting in the downregulation of phosphorylated GSK-3β. Snail and Slug, the necessary regulators of EMT, were inhibited by puerarin as well (39). In summary, these data showed that puerarin may inhibit HCC cell proliferation and migration through alleviating the EMT process.

PTEN, a well-characteristic tumor suppressor gene, has been identified as an effective regulator for EMT in HCC. Some studies have demonstrated that PTEN is a major inhibitor of EMT, which has been confirmed *in vitro* and in experimental models in HCC (41,42). Therefore, identifying drugs that restore PTEN-inhibited EMT might be a potential therapeutic strategy for HCC. Interestingly, our results showed that puerarin also upregulated PTEN expression in HCC cells. In contrast, a PTEN inhibitor reversed the puerarin-induced inhibition of HCC cell proliferation and migration, as well as EMT progression. When HCC appears in the liver, the abnormal expression of various miRNAs is evident. More importantly, miRNAs regulate almost one-third of all human genes. MiR-21 could attenuate the function of PTEN in HCC. Thus, the targeted regulation of miR-21 can increase the tumor suppressive effect of PTEN. Our qRT-PCR results confirmed a negative function of puerarin on miR-21 expression. Moreover, when we increased miR-21 expression through a miR-21 mimic in HCC cells, the inhibition effect of puerarin was abolished.

We conclude that puerarin can inhibit HCC proliferation and metastasis by inhibiting the EMT process. Interestingly, the miR-21/PTEN AKT pathway may mediate the anti-EMT effect of puerarin – and thereby metastasis – in HCC. Thus, puerarin may be a novel HCC suppressor drug.
