# Erratum for: “Puerarin inhibits hepatocellular carcinoma invasion and metastasis through miR-21-mediated PTEN/AKT signaling to suppress the epithelial-mesenchymal transition” [Braz J Med Biol Res (2020) 53(4): e8882]

**DOI:** 10.1590/1414-431X20208882erratum

**Published:** 2020-06-10

Zhou Yuanhttps://orcid.org/0000-0001-9867-0691 ^1*^, Xue Ruifenghttps://orcid.org/0000-0002-4604-5760 ^2*^, Wang Jinglinhttps://orcid.org/0000-0002-3625-2993 ^1^, Ren Haozhenhttps://orcid.org/0000-0001-6275-5052 ^1^

^1^Department of Hepatobiliary Surgery, Affiliated Drum Tower Hospital of Nanjing University Medical School, Nanjing, Jiangsu Province, China

^2^Department of Hepatobiliary Surgery, Nanjing Drum Tower Hospital Clinical College of Nanjing Medical University, Nanjing, Jiangsu Province, China

**Erratum for:** Braz J Med Biol Res | doi: http://10.1590/1414-431X20198882 | PMID: 32294699 | PMCID: PMC7162583

The authors would like to correct the Corresponding authors’ names and emails and the Acknowledgments section of the article “Puerarin inhibits hepatocellular carcinoma invasion and metastasis through miR-21-mediated PTEN/AKT signaling to suppress the epithelial-mesenchymal transition”. The Editors highlight that the information presented in the original version was provided by the authors at submission and revision. In addition, the authors had the opportunity to correct the information during proofreading, before approving for publication.

Correspondence: Haozhen Ren: <renhaozhen1984@163.com> | Jinglin Wang <cw20120817@163.com>

Acknowledgments: This work was supported by grants from the Nanjing Medical Science and Technique Development Foundation (No. QRX17129), the Certificate of China Postdoctoral Science Foundation Grant (2018M642222), and the Jiangsu Province Natural Science Foundation (BK20190114).

*These authors contributed equally to this study.

