# **Erratum 2 for:** “Puerarin inhibits hepatocellular carcinoma invasion and metastasis through miR-21-mediated PTEN/AKT signaling to suppress the epithelial-mesenchymal transition” [Braz J Med Biol Res (2020) 53(4): e8882]

**DOI:** 10.1590/1414-431X2022e8882erratum2

**Published:** 2022-07-13

Yuan Zhou^1*^ https://orcid.org/0000-0001-9867-0691, Ruifeng Xue^2*^ https://orcid.org/0000-0002-4604-5760, Jinglin Wang^1^ https://orcid.org/0000-0002-3625-2993, and Haozhen Ren^1^ https://orcid.org/0000-0001-6275-5052

^1^Department of Hepatobiliary Surgery, Affiliated Drum Tower Hospital of Nanjing University Medical School, Nanjing, Jiangsu Province, China

^2^Department of Hepatobiliary Surgery, Nanjing Drum Tower Hospital Clinical College of Nanjing Medical University, Nanjing, Jiangsu Province, China

Correspondence: Haozhen Ren: <renhaozhen1984@163.com> | Jinglin Wang: <cw20120817@163.com>

*These authors contributed equally to this study.

**Erratum 2 for:** Braz J Med Biol Res | doi: 10.1590/1414-431X20198882 | PMID: 32294699 | PMCID: PMC7162583 

The authors would like to correct Figure 1D, Figure 3A, and Figure 5E that were published incorrectly due to their lack of attention at submission and approval for publication of the article “Puerarin inhibits hepatocellular carcinoma invasion and metastasis through miR-21-mediated PTEN/AKT signaling to suppress the epithelial-mesenchymal transition”.

The fluorescent images of the EdU assay for puerarin in Figure 1D and Figure 5E were wrongly used during the assembly of Figures 1 and 5. The original data obtained on June 28, 2018 were tracked down, and the correct images have been replaced in Figure 1D and Figure 5E, as shown below with the red outline.

The Snail strip image of Huh7 in Figure 3A was accidentally moved during assembly. The original data obtained on August 15, 2018 were tracked down, and the image has been replaced with the correct raw data, as shown below with the red outline.

**Figure 1:**
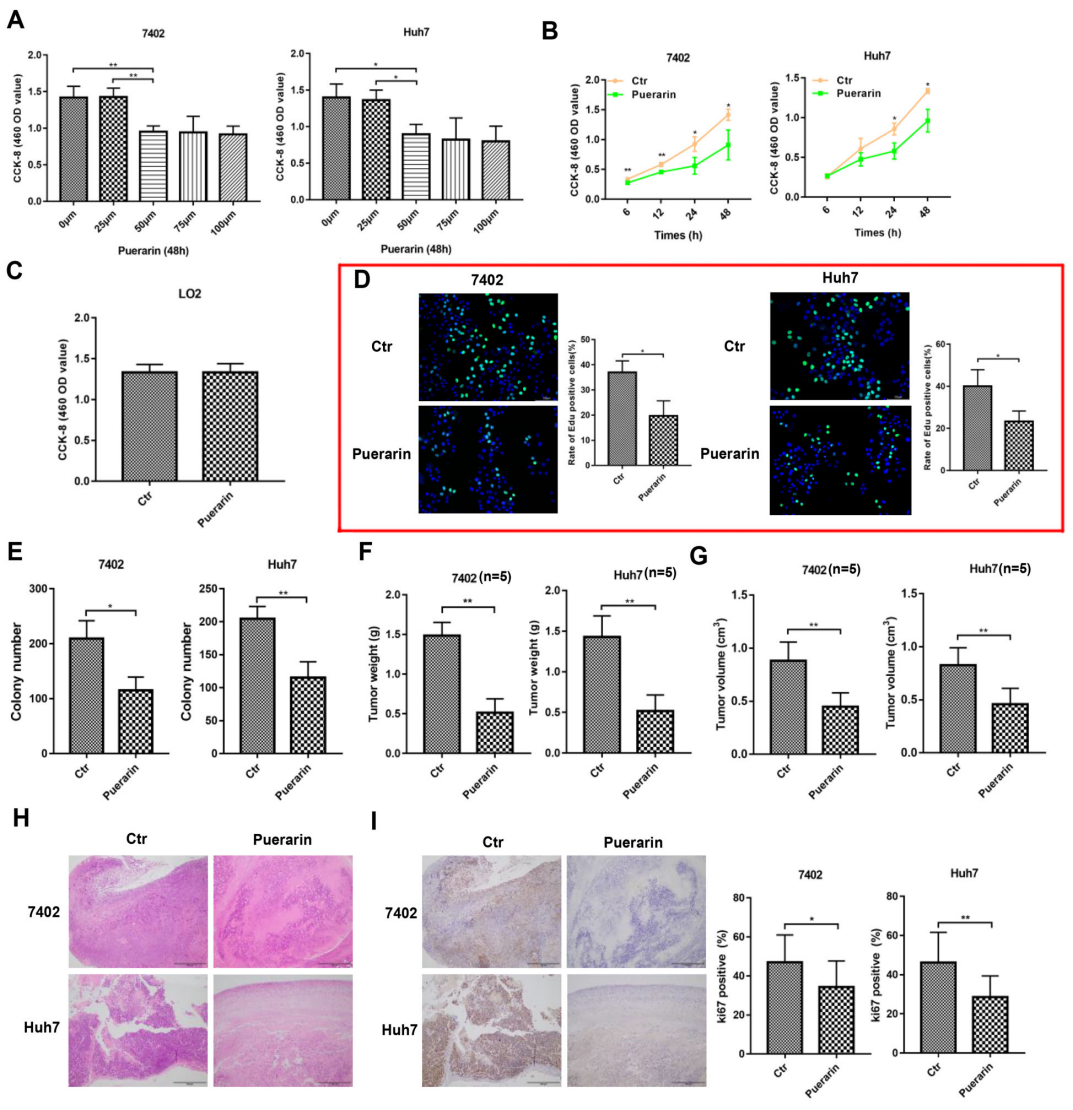
Effects of puerarin on hepatocellular carcinoma (HCC) proliferation and growth. **A** and **B**, CCK-8 assays of HCC cells treated by puerarin at different concentrations and time points. **C**, CCK-8 assays of L02 cells treated with puerarin at the concentration of 50 nM. **D** and **E**, EdU assay and liquid colony formation analysis of HCC cells were conducted to detect the anti-tumor effects of puerarin (magnification bar: 100 μm). **F** and **G**, Colony number and tumor weight of HCC cells treated with puerarin or control. **H**, H&E staining was performed on serial sections of mouse tumors induced from HCC cells (magnification bar: 100 μm). **I**, Immunohistochemistry analysis of Ki67 expression and qualification of Ki67-positive cells in mouse tumors (magnification bar: 100 μm). At least three independent experiments with similar results were done. Data are reported as means±SD. *P<0.05, **P<0.01 (*t*-test). Ctr: control.

**Figure 3:**
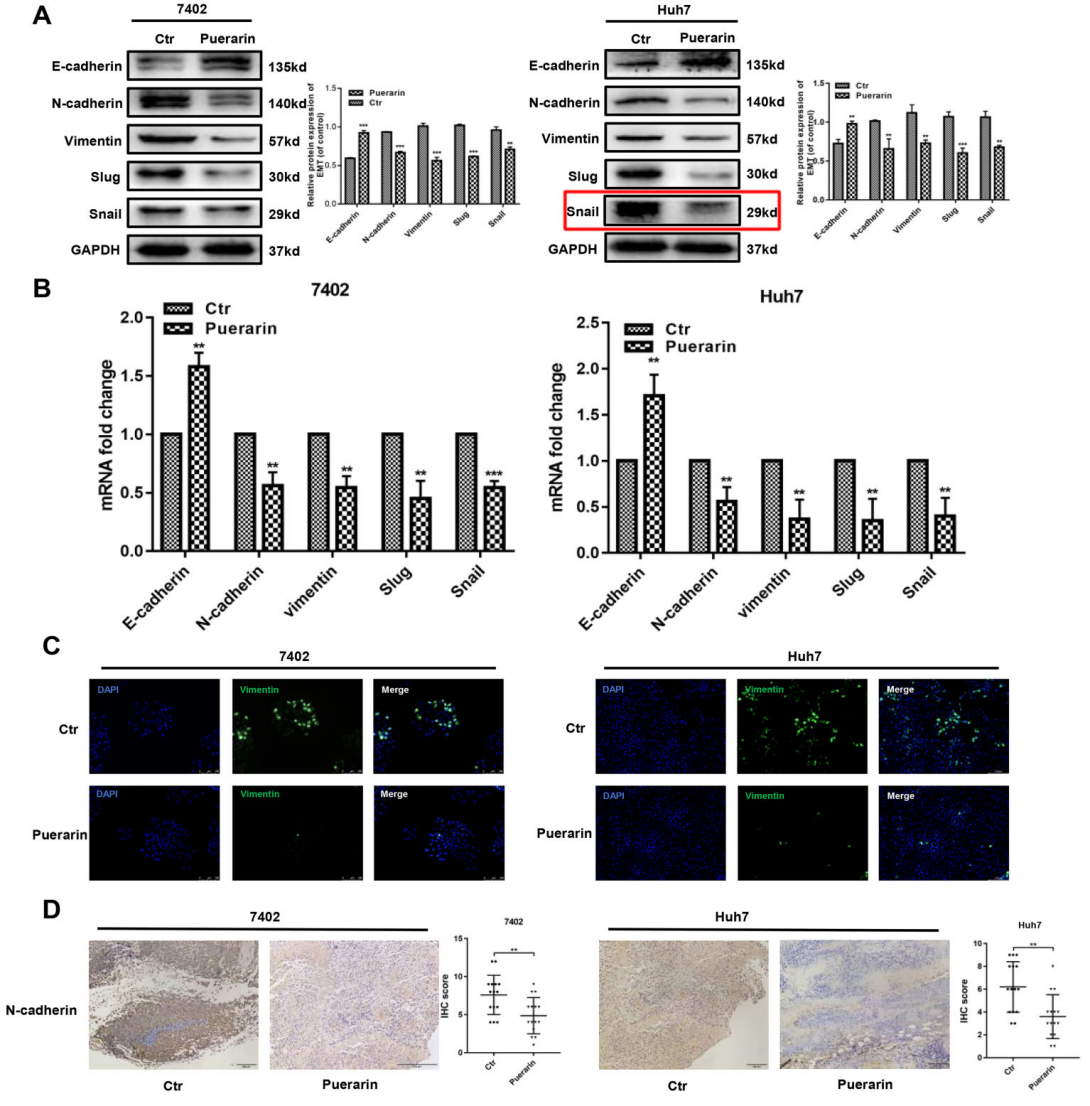
Effect of puerarin on epithelial-mesenchymal transition (EMT) genes. **A**, Western blotting analysis and **B**, qRT-PCR analysis were used to detect the expression of EMT markers in puerarin-treated hepatocellular carcinoma (HCC) cells. **C**, Immunofluorescence analysis of vimentin expression (magnification bars: 100 μm). **D**, Immunohistochemistry analysis (IHC) of N-cadherin expression was performed on hepatocellular tumors (magnification bars: 100 μm). Statistical analyses of the staining intensity of N-cadherin between different groups are shown. Data are reported as means±SD. **P<0.01, ***P<0.001 (*t*-test). Ctr: control.

**Figure 5:**
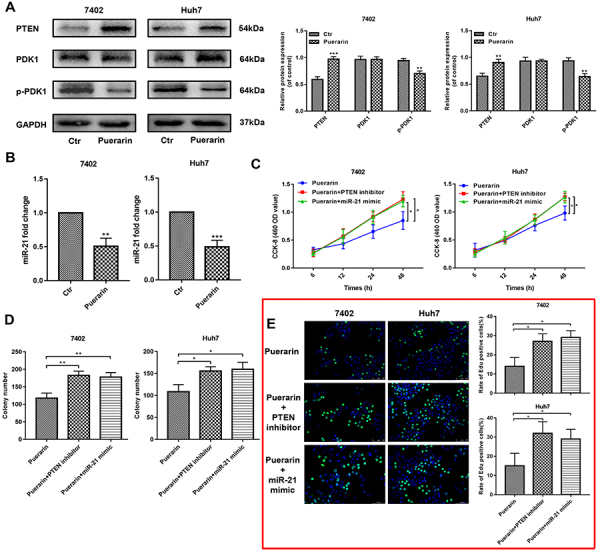
miR-21 mimic and phosphate and tension homolog (PTEN) inhibitor reversed the anti-tumor effects of puerarin in hepatocellular carcinoma (HCC) cells. **A**, Western blotting analysis was used to detect the PTEN expression and its downstream protein expression. **B**, qRT-PCR analysis confirmed the expression change of miR-21 in puerarin treated HCC cells. **C**, CCK-8 assays of treated HCC cells with miR-21 mimic and PTEN inhibitor at different time points. **D** and **E**, Liquid colony formation analysis and EdU assay of miR-21-infected and PTEN-inhibited HCC cells under the treatment of puerarin (magnification bars: 100 μm). Data are reported as means±SD. *P<0.05, **P<0.01, ***P<0.001 (*t*-test). Ctr: control.

